# Overexpression of PODXL/ITGB1 and BCL7B/ITGB1 accurately predicts unfavorable prognosis compared to the TNM staging system in postoperative pancreatic cancer patients

**DOI:** 10.1371/journal.pone.0217920

**Published:** 2019-06-05

**Authors:** Keisuke Taniuchi, Mutsuo Furihata, Seiji Naganuma, Masahiko Sakaguchi, Toshiji Saibara

**Affiliations:** 1 Department of Gastroenterology and Hepatology, Kochi Medical School, Kochi University, Nankoku, Kochi, Japan; 2 Department of Endoscopic Diagnostics and Therapeutics, Kochi Medical School, Kochi University, Nankoku, Kochi, Japan; 3 Department of Pathology, Kochi Medical School, Kochi University, Nankoku, Kochi, Japan; 4 Integrated Center for Advanced Medical Technologies, Kochi Medical School, Kochi University, Nankoku, Kochi, Japan; 5 Cancer Prevention and Control Division, Kanagawa Cancer Center Research Institute, Yokohama, Kanagawa, Japan; Institut national de la recherche scientifique, CANADA

## Abstract

We previously reported that overexpression of PODXL, BCL7B, and ARHGEF4 in pancreatic cancer tissue is correlated with pancreatic cancer-related survival. The aim of this study was to investigate the use of PODXL, BCL7B, ARHGEF4, and the integrin family member ITGB1 as useful markers for the prognosis of postoperative pancreatic cancer patients in comparison with tumor size and the tumor node metastasis (TNM) staging system. Immunohistochemistry was performed using an anti-ITGB1 antibody on 102 samples of pancreatic cancer tissue surgically resected at the University of Kochi Medical School Hospital and the Matsuyama Shimin Hospital. Univariate Cox proportional hazards regression analysis showed that TNM stage and overexpression of PODXL, BCL7B, and ITGB1 were correlated with postoperative survival. However, tumor size was not significantly associated with postoperative prognosis of pancreatic cancer compared to these features. Multivariate Cox proportional hazards regression analysis showed that the overexpression of both PODXL and ITGB1 and overexpression of both BCL7B and ITGB1 increased the hazard ratio (6.27, 95% confidence interval [CI] 2.58–15.21; and 3.93, 95% CI 1.74–8.91, respectively) compared to that of TNM stage (IIA and IIB vs. III and IV; 3.05, 95% CI 1.25–7.42). These results imply that the combination of PODXL with ITGB1 and the combination of BCL7B with ITGB1 accurately predicted the postoperative outcomes of pancreatic cancer patients, and they were superior compared to the TNM staging system. The combination of PODXL with ITGB1 would be particularly useful, as it was the most highly correlated with postoperative outcomes. Importantly, the present results are useful to determine which adjuvant therapy should be selected.

## Introduction

Pancreatic ductal adenocarcinoma (PDAC) is one of the most aggressive tumors, and the prognosis is poor, with 1- and 5-year survival rates of only 20% and 6%, respectively [1, 2]. Complete tumor resection is the only potential treatment for PDAC that results in a complete cure [3]. Since about half of PDAC patients are diagnosed with end-stage disease, 35% with localized unresectable disease, and 20% with potentially resectable disease, surgery is not always suitable [4]. Neoadjuvant therapies for PDAC patients with borderline resectable and locally-advanced disease have been proposed to achieve tumor down-staging to a subsequent potentially resectable tumor [5]. Additionally, postoperative adjuvant chemotherapies improve both PDAC-related and disease-free survival. A phase III trial (PRODIGE24) recently demonstrated that adjuvant chemotherapy with a modified FOLFIRINOX regimen (5-fluorouracil, leucovorin, irinotecan, and oxaliplatin) significantly increases overall survival compared with gemcitabine for 24 weeks after resection of PDAC [6]. However, there are no reliable biomarkers to gauge the response to neoadjuvant and/or adjuvant therapies prior to the initiation of the therapies [7].

The Union for International Cancer Control (UICC) tumor node metastasis (TNM) staging system for PDAC is currently based on histologically determined tumor size, tumor invasion to the celiac axis and superior mesenteric artery, involvement of regional lymph nodes, and the occurrence of metastatic spread to other organs [8]. The 5-year survival of PDAC patients treated with resection with or without adjuvant therapies is 16–25% for stage IIA and 8–10% for stage IIB [9], indicating that PDAC treated at stage IIA has better outcomes compared to that at stage IIB. Moreover, a PDAC size >20 mm correlates with postoperative outcomes and is an independent predictor of poor postoperative prognosis [9]. Thus, the UICC TNM staging system for resected PDAC is a useful predictor of postoperative prognosis, but more reliable prognostic predictors that can discriminate PDAC patients with stage IIA and IIB into two prognosis groups (longer disease-free survival and/or better PDAC-related survival vs. shorter disease-free survival and/or poor PDAC-related survival) are necessary for clinical decision-making.

We previously reported that knockdown of the podocalyxin-like protein (PODXL), B-cell CLL/lymphoma 7B (BCL7B), and Rho guanine nucleotide exchange factor 4 (ARHGEF4) by small interfering RNAs inhibits the *in vitro* motility and invasiveness of PDAC cells by decreasing cell protrusions [10, 11, 12]. Overexpression of PODXL, BCL7B, and ARHGEF4 in PDAC tissue is significantly correlated with postoperative prognosis [10, 11, 12]. Integrin β1 (*ITGB1*) mRNA binds to insulin-like growth factor-2 mRNA-binding protein 3 (IGF2BP3) in PDAC cells [13]. Locally translated IGF2BP3-bound mRNAs in PDAC cell protrusions induce the formation of those protrusions, thereby promoting invasiveness and metastasis [13, 14]. Thus, these reports suggest that ITGB1 protein concentrated in protrusions may promote the cell motility and invasiveness of PDAC cells.

In the present study, we investigated the use of PODXL, BCL7B, ARHGEF4, and ITGB1 as useful markers for the prognosis of postoperative PDAC patients in comparison with tumor size and the TNM staging system. We showed that a combination of PODXL with ITGB1 and a combination of BCL7B with ITGB1 predicted the postoperative outcomes of PDAC patients better than tumor size and the TNM staging system.

## Results

### ITGB1 expression in PDAC tissue samples

**Fig 1:**
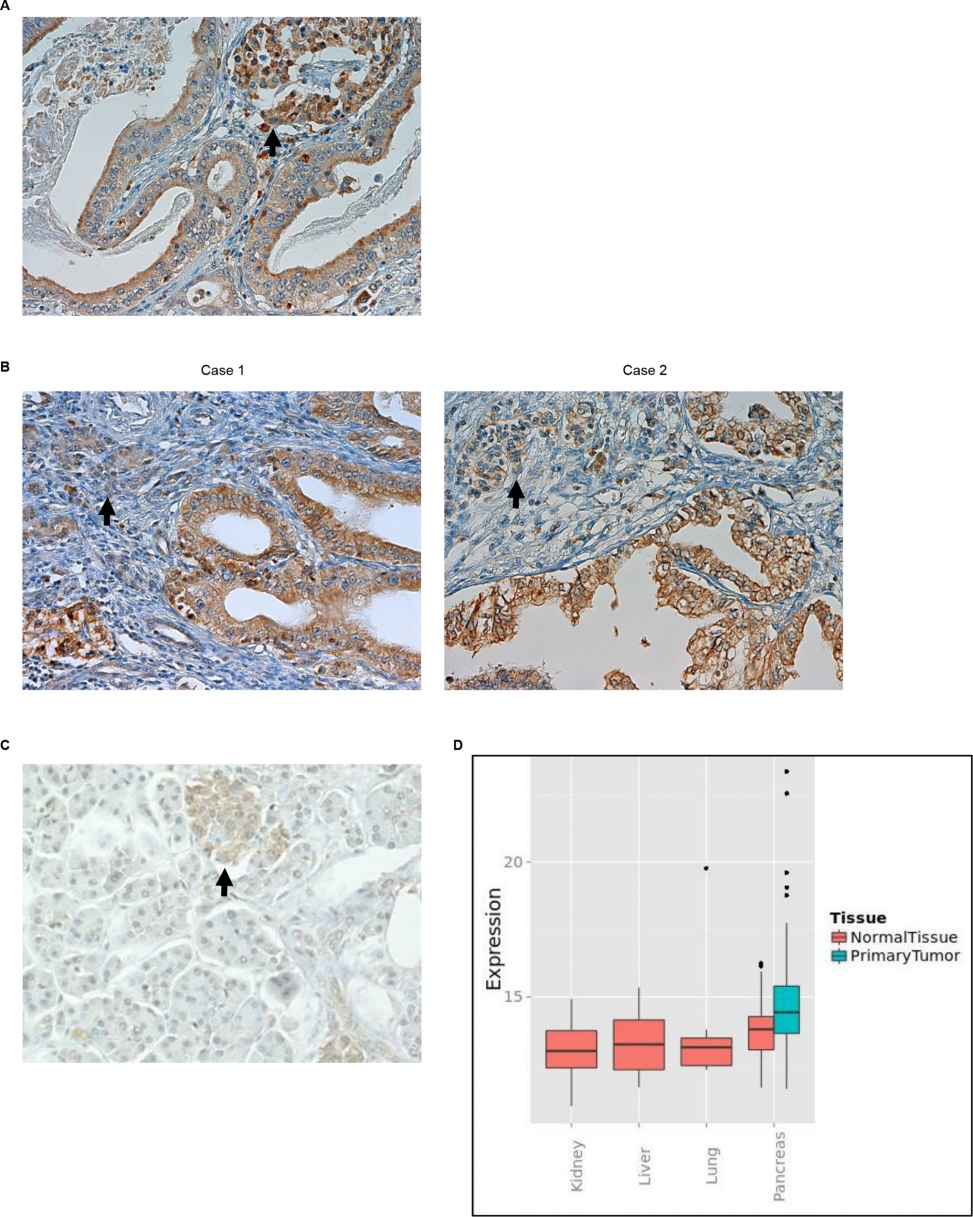
Immunohistochemistry with anti-ITGB1 antibody. (A) Representative immunohistochemical staining of PDAC tissue using anti-ITGB1 antibody showing low expression of ITGB1. Arrow, islet of Langerhans. Magnification: ×200. (B) Immunohistochemical staining of two PDAC tissue samples using anti-ITGB1 antibody showing high expression of ITGB1. Arrow, islet of Langerhans. Magnification: ×200. (C) Expression of ITGB1 in normal pancreas. Arrow, islet of Langerhans. Magnification: ×200. (D) mRNA expression distribution of *ITGB1* between pancreatic tumor tissue and normal organ tissues including kidney, liver, lung, and pancreas.

Immunohistochemical analysis showed that ITGB1 was present in all 102 PDAC cases, and scores of immunostaining were classified into a low-expressing ITGB1 group (67.6%, Fig 1A) and high-expressing ITGB1 group (32.4%, Fig 1B) (Table 1). Although the islets of Langerhans in normal pancreas were stained with anti-ITGB1 antibody as well as those in PDAC tissues, ITGB1 staining was not found in normal pancreatic ducts (Fig 1C). Similarly, an association analysis of PDAC tissue and normal organ tissues including kidney, liver, lung, and pancreas using the Metabolic gEne RApid Visualizer (MERAV; http://merav.wi.mit.edu/) [15] showed that *ITGB1* mRNA was markedly upregulated in PDAC tissue (Fig 1D).

**Table 1:** Summary of characteristics of 102 patients with pancreatic cancer.

Caracteristics	Percentage (%)		Charasteristics	Percentage (%)	
**Age at surgery**			Distant metastasis*		
40–50	3.9	[n = 4]	M0	96.1	[n = 98]
50–60	16.7	[n = 17]	M1	3.9	[n = 4]
60–70	31.4	[n = 32]	**Histology**†		
70–80	40.2	[n = 41]	PanIN	2.0	[n = 2]
> 80	7.8	[n = 8]	well	29.4	[n = 30]
**Gender**			moderate	58.8	[n = 60]
Male	54.9	[n = 56]	poor	9.8	[n = 10]
Female	45.1	[n = 46]	**Venous invasion**†		
Stage*			v0	55.4	[n = 57]
0	2.0	[n = 2]	v1	30.7	[n = 31]
IA	3.9	[n = 4]	v2	10.9	[n = 11]
IB	7.8	[n = 8]	v3	3.0	[n = 3]
IIA	31.4	[n = 32]	**Lymphatic invasion**†		
IIB	49.0	[n = 50]	ly0	42.6	[n = 43]
III	2.0	[n = 2]	ly1	33.6	[n = 34]
IV	3.9	[n = 4]	ly2	19.9	[n = 21]
Primary tumor*			ly3	3.9	[n = 4]
Tis	2.0	[n = 2]			
T1	5.9	[n = 6]	**Adjuvant therapy**		
T2	14.6	[n = 15]	Chemotherapy	44.1	[n = 45]
T3	75.5	[n = 77]	Radiation therapy	3.9	[n = 4]
T4	2.0	[n = 2]	Chemoradiation therapy	36.3	[n = 37]
Regional lymph nodes*					
N0	45.1	[n = 46]	**ITGB1 expression**		
N1	54.9	[n = 56]	Low	67.6	[n = 69]
			High	32.4	[n = 33]

*, Classified according to the classification of International Union against Cancer

†, Classified according to the classification of pancreatic cancer of Japan Pancreas Society; PanIN, pancreatic intraepithelial neoplasia.

### Associations of ITGB1 overexpression with clinicopathological factors and with prognosis

Of 102 PDAC patients, 86 had received adjuvant chemotherapy with gemcitabine or S-1, or radiation therapy or chemoradiation therapy after resection of the PDAC (Table 1). There was no significant correlation found between adjuvant therapy and PDAC patient prognosis (S1 Fig and S1 Table).

The association of ITGB1 expression levels in PDAC tissue with clinicopathological variables is shown in Table 2. No significant clinicopathological variables were correlated with the ITGB1 expression level.

**Table 2:** Correlation between ITGB1 expression and clinicopathological parameters.

	ITGB1 expression				*P*
	Low		High		
Stage*	percentage (%)				0.356
0	2.9	[n = 2]	0	[n = 0]	
IA	2.9	[n = 2]	6.0	[n = 2]	
IB	10.1	[n = 7]	3.0	[n = 1]	
IIA	30.4	[n = 21]	33.3	[n = 11]	
IIB	49.3	[n = 34]	48.6	[n = 16]	
III	2.9	[n = 2]	0	[n = 0]	
IV	1.5	[n = 1]	9.1	[n = 3]	
Primary tumor*					0.878
Tis	2.9	[n = 2]	0	[n = 0]	
T1	5.8	[n = 4]	6.0	[n = 2]	
T2	13.0	[n = 9]	18.2	[n = 6]	
T3	75.4	[n = 52]	75.8	[n = 25]	
T4	2.9	[n = 2]	0	[n = 0]	
Regional lymph nodes*					0.675
N0	43.5	[n = 30]	48.6	[n = 16]	
N1	56.5	[n = 39]	51.4	[n = 17]	
Distant metastasis*					0.0982
M0	98.5	[n = 68]	90.9	[n = 30]	
M1	1.5	[n = 1]	9.1	[n = 3]	
**Histology**†					0.172
PanIN	2.9	[n = 2]	0	[n = 0]	
well	34.8	[n = 24]	18.2	[n = 6]	
moderate	55.1	[n = 38]	66.7	[n = 22]	
poor	7.2	[n = 5]	15.1	[n = 5]	
**Venous invasion**†					0.766
v0 + v1	87.0	[n = 60]	84.9	[n = 28]	
V2 + v3	13.0	[n = 9]	15.1	[n = 5]	
**Lymphatic invasion**†					0.372
ly0 + ly1	88.4	[n = 61]	81.8	[n = 27]	
ly2 + ly3	11.6	[n = 8]	18.2	[n = 6]	

*, Classified according to the classification of International Union against Cancer

†, Classified according to the classification of pancreatic cancer of Japan Pancreas Society; PanIN, pancreatic intraepithelial neoplasia.

**Fig 2:**
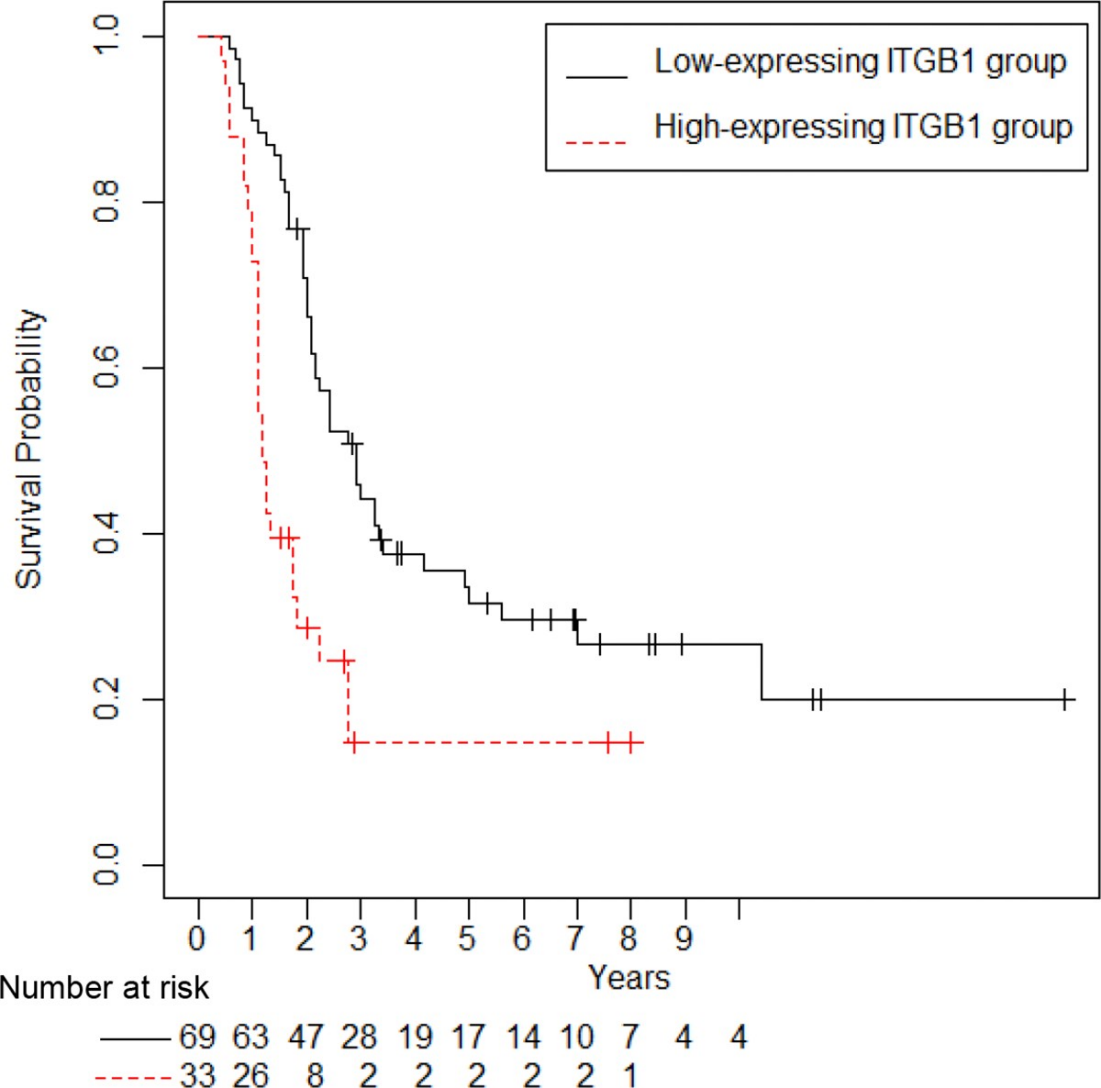
Correlation between high expression of ITGB1 and poor outcomes in PDAC patients. Kaplan-Meier analysis of postoperative survival according to ITGB1 expression is shown.

Kaplan-Meier curves showed that the postoperative survival time for PDAC patients with ITGB1 overexpression was significantly shorter than that of PDAC patients with low ITGB1 expression (P < 0.001; Fig 2, S2 Table). We examined the prognostic value of ITGB1 expression in subgroups stratified by UICC TNM stage, age, gender, tumor size, differentiation grade, lymphatic invasion, venous invasion, and intrapancreatic nerve invasion. Univariate Cox regression analysis revealed that UICC TNM stage, high ITGB1 expression, tumor size, and venous invasion served as independent prognostic factors (Table 3). Furthermore, multivariate analysis revealed that UICC TNM stage and high ITGB1 expression were independent factors of worse PDAC-related survival (Table 3). These results suggested that ITGB1 is an independent predictor of worse postoperative survival of PDAC.

**Table 3:** Univariate and multivariate analysis of prognostic factors for overall survival.

	Overall survival			
	Univariate		Multivariate	
	HR (95% CI)	*P*	HR (95% CI)	*P*
Stage*				
0 + IA + IB	0.21 (0.07–0.59)	0.002	0.21 (0.07–0.60)	0.003
IIA + IIB	Reference		Reference	
III + IV	2.56 (1.09–5.98)	0.029	2.63 (1.12–6.20)	0.001
**Age at surgery**	1.02 (0.99–1.04)	0.110	1.02 (0.99–1.05)	0.065
**Gender**				
Female	Reference		Reference	
Male	1.10 (0.69–1.76)	0.667	1.14 (0.72–1.83)	0.559
**ITGB1 expression**				
Low	Reference		Reference	
High	2.50 (1.52–4.12)	<0.0001	2.25 (1.36–3.73)	0.001
**Diameter of primary tumor**				
<2 cm	Reference			
2.0–3.0 cm	1.34 (0.58–3.05)	0.488		
>3 cm	1.71 (0.76–3.87)	0.192		
**Histology**†				
PanIN + well-differentiated	Reference			
Moderately + poorly-differentiated	1.38 (0.84–2.26)	0.196		
**Lymphatic invasion**†				
ly0 + ly1	Reference			
ly2 + ly3	1.26 (0.75–2.14)	0.373		
**Venous invasion**†				
v0 + v1	Reference			
v2 + v3	1.92 (1.03–3.59)	0.038		
**Intrapancreatic nerve invasion†**				
n0 + n1	Reference			
** n2 + n3**	**1.50 (0.94–2.37)**	**0.083**		

*, Classified according to the classification of International Union against Cancer

†, Classified according to the classification of pancreatic cancer of Japan Pancreas Society

### Ability of PODXL, BCL7B, ARHGEF4, and ITGB1 to predict prognosis compared to UICC TNM stage and tumor size

**Fig 3:**
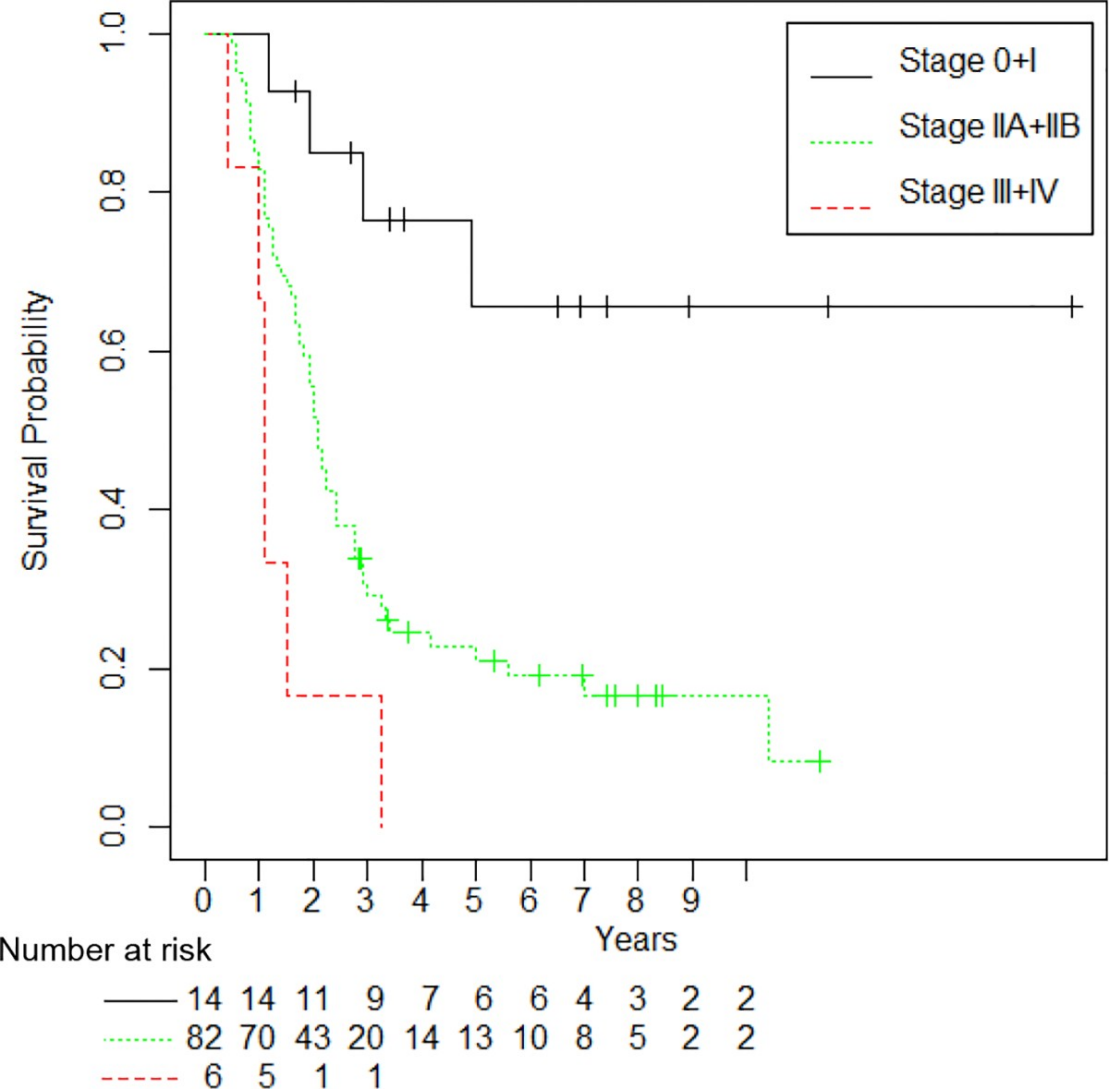
Kaplan-Meier curves for PDAC-related survival according to UICC TNM stage. Kaplan-Meier analysis of PDAC-specific survival by UICC TNM stage is shown.

We investigated the abilities of PODXL, BCL7B, ARHGEF4, and ITGB1 to predict prognosis in PDAC in comparison with UICC TNM stage and tumor size. The PDAC-related survival time for postoperative PDAC patients according to UICC TNM stage is shown in Fig 3, and UICC TNM stage did predict the prognosis of PDAC patients. To analyze the ability of PODXL, BCL7B, ARHGEF4, and ITGB1 to predict postoperative prognosis, we used the immunostaining scores of PODXL, BCL7B, and ARHGEF4 in the present 102 PDAC tissue samples that we previously reported [10, 11, 12]. Univariate analysis using Cox proportional hazards regression analysis showed that the accuracy of the immunostaining scores of PODXL, BCL7B, ARHGEF4, and ITGB1 to predict prognosis was almost the same as that of UICC TNM staging and better than that of tumor size (HR: 2.89, 95% CI: 1.78–4.68 for PODXL; HR: 2.27, 95% CI: 1.37–3.74 for BCL7B; HR: 2.39, 95% CI: 1.45–3.93 for ARHGEF4; HR: 2.50, 95% CI: 1.52–4.12 for ITGB1; HR: 2.56, 95% CI: 1.09–5.98 for UICC TNM stage III-IV; and HR: 1.72, 95% CI: 0.75–3.88 for tumor size) (Table 4). Multivariate analysis using a backwards and a forwards selection procedure showed that the final model included UICC TNM stage, PODXL, BCL7B, and ITGB1, which were the most independent variables that predicted prognosis accurately (Table 5).

**Table 4:** Univariate analysis using the Cox proportional hazards regression analysis.

	HR (95% CI)
**Age at surgery**	1.02(0.99–1.04)
**Gender**	1.10(0.69–1.76)
Stage*	
0, IA, IB	0.21 (0.07–0.59)
IIA, IIB	Reference
III, IV	2.56 (1.09–5.98)
**Histology**†	1.38 (0.84–2.26)
**Venous invasion**†	1.92 (1.03–3.59)
**Lymphatic invasion**†	1.26 (0.75–2.14)
**Intrapancreatic nerve invasion**†	1.50 (0.94–2.37)
**Tumor size**	
< 2 cm	Reference
2.0–3.0 cm	1.34 (0.59–3.06)
> 3 cm	1.71 (0.76–3.87)
**PODXL expression**	2.89 (1.78–4.68)
**BCL7B expression**	2.27 (1.37–3.74)
**ARHGEF4 expression**	2.39 (1.45–3.93)
**ITGB1 expression**	2.50 (1.52–4.12)

*, Classified according to the classification of International Union against Cancer

†, Classified according to the classification of pancreatic cancer of Japan Pancreas Society.

**Table 5:** Multivariate analysis using a backwards and a forwards selection procedure.

	HR (95% CI)	P
Stage*		
0, IA, IB	0.20 (0.07–0.58)	0.003
IIA, IIB	Reference	
III, IV	4.05 (1.65–9.94)	0.002
**PODXL expression**	2.34 (1.37–4.01)	0.002
**BCL7B expression**	1.86 (1.01–3.41)	0.04
**ARHGEF4 expression**	1.71 (0.93–3.13)	0.09
**ITGB1 expression**	1.73 (1.02–2.94)	0.04

*, Classified according to the classification of International Union against Cancer.

### Ability of the combination of PODXL, BCL7B, and ITGB1 to predict prognosis of all PDAC patients

Among PODXL, BCL7B, ARHGEF4, and ITGB1, we investigated the potential of using a combination of two proteins for prediction of prognosis in resected PDAC in comparison with each of PODXL, BCL7B, ARHGEF4, ITGB1, UICC TNM stage, age, gender, tumor size, differentiation grade, lymphatic invasion, venous invasion, and intrapancreatic nerve invasion. The variable selection procedure showed that the final model included UICC TNM stage, the combination of PODXL with ITGB1, and the combination of BCL7B with ITGB1 (Table 6). PODXL, BCL7B, ARHGEF4, ITGB1, or tumor size were not included in the final model of the multivariate analysis. The abilities of the combination of PODXL with ITGB1 and of the combination of BCL7B with ITGB1 to predict prognosis of resected PDACs were superior to UICC TNM stage (HR: 6.27, 95% CI: 2.58–15.21 for the combination of PODXL with ITGB1; HR: 3.93, 95% CI: 1.74–8.91 for the combination of BCL7B with ITGB1; and HR: 3.05, 95% CI: 1.25–7.42 for UICC TNM stage) (Table 6).

**Table 6:** Multivariate analysis using the Cox proportional hazards regression model.

	HR (95% CI)	P
Stage*		
0, IA, IB	0.25 (0.09–0.70)	0.009
IIA, IIB	Reference	
III, IV	3.05 (1.25–7.42)	0.014
**ARHGEF4 expression**	2.52 (1.28–5.00)	0.007
**ARHGEF4 expression and intrapancreatic nerve invasion**	2.97 (1.36–6.49)	0.006
**ARHGEF4 and ITGB1 expression**	0.22 (0.08–0.59)	0.003
**PODXL and ITGB1 expression**	6.27 (2.58–15.2)	< 0.001
**BCL7B and ITGB1 expression**	3.93 (1.74–8.91)	0.001

*, Classified according to the classification of International Union against Cancer.

**Fig 4:**
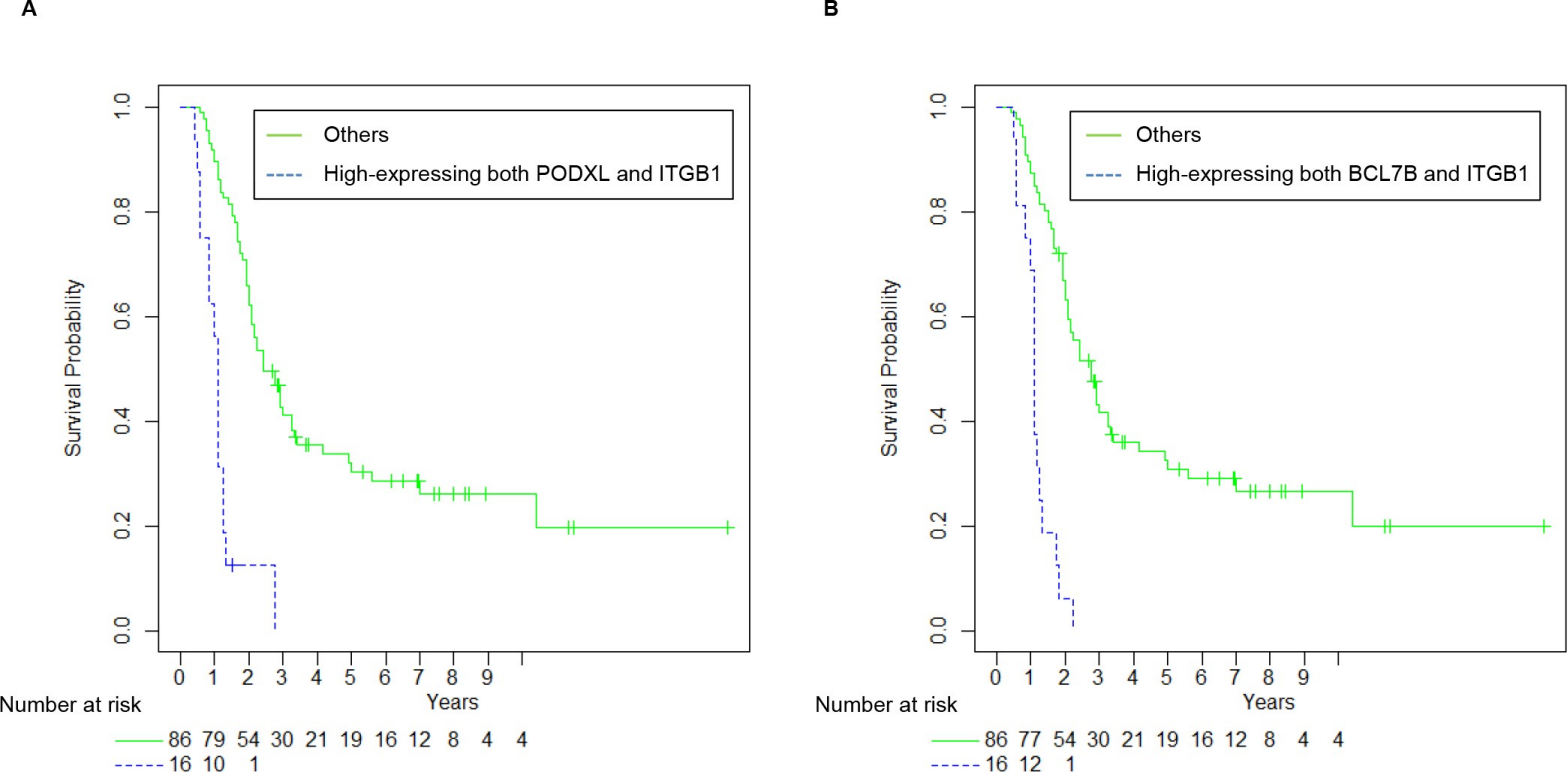
Correlation between high expression of two protein combinations and poor outcomes in all PDAC patients. (A, B) Kaplan-Meier analysis of postoperative survival according to (A) the combination of PODXL with ITGB1 and (B) the combination of BCL7B with ITGB1 in all PDAC patients.

Kaplan-Meier curves confirmed that overexpression of both PODXL and ITGB1 (n = 16, including 7 patients at stage IIA, 8 patients at stage IIB, and 1 patient at stage IV) and overexpression of both BCL7B and ITGB1 (n = 16, including 5 patients at stage IIA, 9 patients at stage IIB, and 2 patients at stage IV) accurately predicted the prognosis of the resected PDAC patients (P < 0.001; Fig 4A and 4B). The 3-year and 5-year survival rates of UICC TNM stage III and IV were 16.7% (95% CI: 3.0–99) and 0% (Table 7). The 3-year survival rate of the high-expressing group of both PODXL and ITGB1 was 0%, and that of the low-expressing group was 41.2% (95% CI: 31.6–53.6) (Table 7). The 5-year survival rate of the low-expressing group of both PODXL and ITGB1 was 30.4% (95% CI: 21.4–43.1) (Table 7). The 3-year survival rate of the high-expressing group of both BCL7B and ITGB1 was 0%, and that of the low-expressing group was 41.8% (95% CI: 32.1–54.4) (Table 7). The 5-year survival rate of the low-expressing group of both BCL7B and ITGB1 was 30.8% (95% CI: 21.8–43.7) (Table 7). The median survival times of UICC TNM stage III and IV, the high-expressing group of both PODXL and ITGB1, and the high-expressing group of both BCL7B and ITGB1 were 13 months (95% CI: 12-NA), 13 months (95% CI: 10–16), and 13 months (95% CI: 12–21), respectively (Table 7). These results suggest that the combination of PODXL with ITGB1 is the best predictor of postoperative outcomes for PDAC patients.

**Table 7:** The 3-year and 5-year survival rates and median survival times.

	n	Survival rate (95% CI) (%)		Median survival time (95%CI) (month)
		3-year	5-year	
Stage*				
0, IA, IB	14	76.6 (56.5–100)	65.7 (42.7–100)	Not reached (59- NA)
IIA, IIB	82	29.3 (20.5–41.7)	21.0 (13.3–33.3)	25 (22–33)
III, IV	6	16.7 (3–99)	0	13 (12-NA)
**ARHGEF4 and ITGB1 expression**				
Both high expression	15	0	0	15 (13-NA)
Others	87	39.4 (30.1–51.5)	29.0 (20.4–41.3)	29 (24–39)
**PODXL and ITGB1 expression**				
Both high expression	16	0	0	13 (10–16)
Others	86	41.2 (31.6–53.6)	30.4 (21.4–43.1)	29 (25–40)
**BCL7B and ITGB1 expression**				
Both high expression	16	0	0	13 (12–21)
Others	86	41.8(32.1–54.4)	30.8 (21.8–43.7)	33 (25–41)

*, Classified according to the classification of International Union against Cancer.

### Ability of the combination of PODXL, BCL7B, and ITGB1 to predict prognosis of PDAC patients at UICC TNM stage IIA and IIB

**Fig 5:**
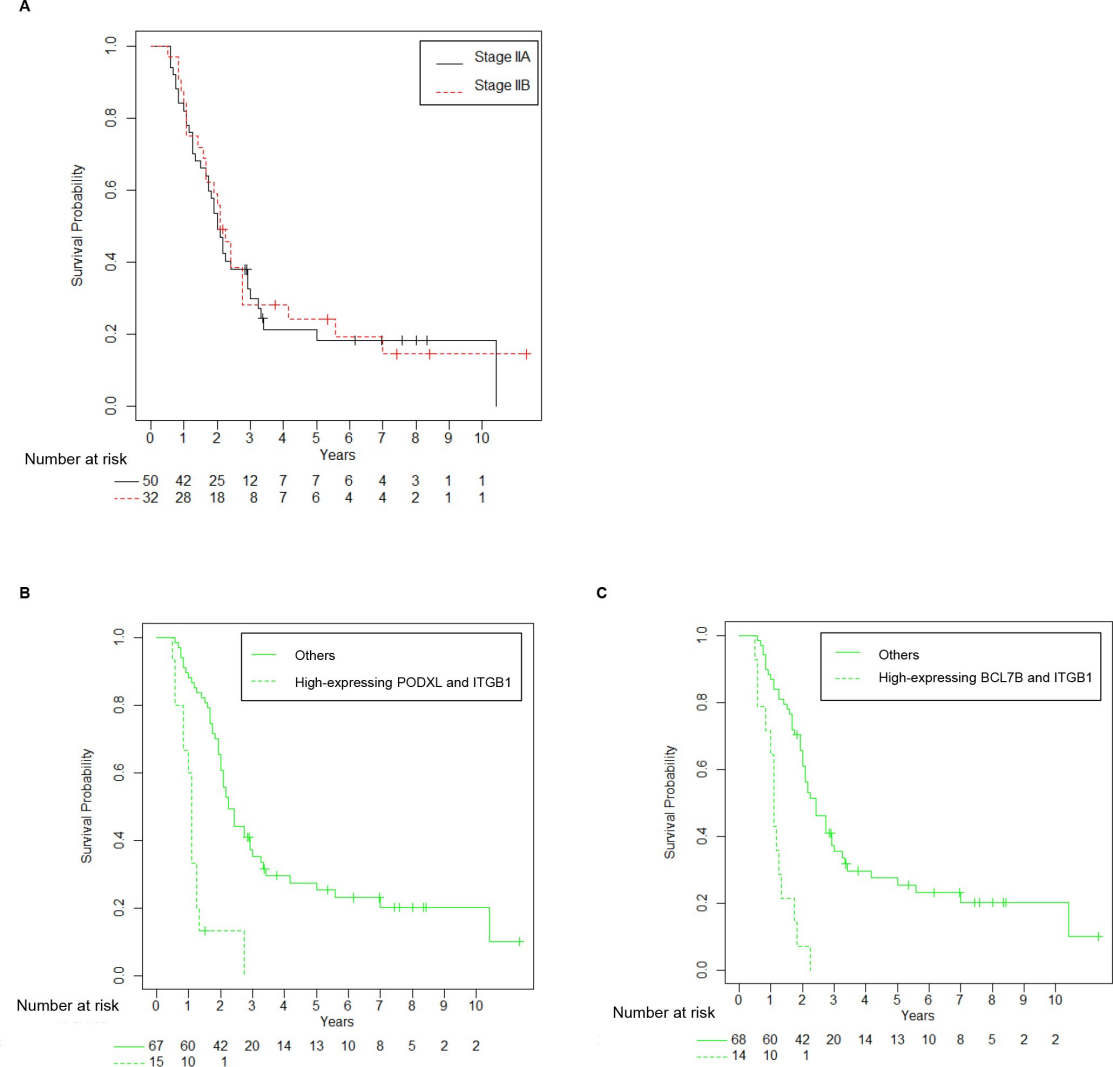
Correlation between high expression of two protein combinations and poor outcomes in PDAC patients at stage IIA and IIB. (A) Kaplan-Meier analysis of PDAC-related survival of patients with stage IIA and IIB tumors. The dashed lines represent the lower and upper limits of the 95% confidence interval. (B, C) Kaplan-Meier analysis of postoperative survival according to (B) the combination of PODXL with ITGB1 and (C) the combination of BCL7B with ITGB1 in stage IIA and IIB PDAC patients.

We focused on the ability of these combinations to predict the postoperative prognosis of PDAC patients at UICC TNM stage IIA and IIB. There were no differences in postoperative survival times between PDAC patients at stage IIA and PDAC patients at stage IIB (Fig 5A). Kaplan-Meier curves showed that the combination of PODXL with ITGB1 and the combination of BCL7B with ITGB1 significantly correlated with surgical outcomes and poor prognosis in UICC TNM stage II PDAC patients (P < 0.001; Fig 5B and 5C). The median survival times of the high-expressing group of both PODXL and ITGB1 at UICC TNM stage II and other PDACs at UICC TNM stage II were 13 months (95% CI: 10–16) and 27 months (95% CI: 24–36), respectively (Table 8). The median survival times of the high-expressing group of both BCL7B and ITGB1 at UICC TNM stage II and other PDACs of both BCL7B and ITGB1 at UICC TNM stage II were 13 months (95% CI: 12–22) and 29 months (95% CI: 25–36), respectively (Table 8). These results suggest that the combination of PODXL with ITGB1 and the combination of BCL7B with ITGB1 are useful to predict the postoperative outcomes of PDAC patients at UICC TNM stage IIA and IIB.

**Table 8:** The 3-year and 5-year survival rates and median survival times at stage II*.

	n	Survival rate (95% CI) (%)		Median survival time (95%CI) (month)
		3-year	5-year	
**PODXL and ITGB1 expression**				
Both high expression	15	0	0	13 (10–16)
Others	67	35.3 (25.1–49.6)	25.4 (16.2–39.8)	27 (24–36)
**BCL7B and ITGB1 expression**				
Both high expression	14	0	0	13 (12–22)
Others	68	35.4 (25.2–49.8)	27.6 (18.1–42.0)	29 (25–36)

*, Classified according to the classification of International Union against Cancer.

## Discussion

The present study showed that high ITGB1 expression is closely associated with poor prognosis of resected PDAC patients, similar to what has been previously published regarding PODXL and BCL7B [10, 11]. The immunohistochemical scores of ITGB1 were not statistically correlated with the clinicopathological variables, but univariate and multivariate Cox regression analyses revealed that high ITGB1 expression was an independent predictor of worse survival outcomes. Consistent with our results, high ITGB1 expression is significantly associated with poor outcomes as well as progression and metastasis in PDAC [12, 16, 17, 18]. Our results indicate that ITGB1 may be a determinant of poor prognosis of PDAC patients that is functionally associated with cell migration, invasion, and/or metastasis.

UICC TNM stage IIB is the most common, and the majority of stage IIB PDAC patients undergo surgery [19, 20]. The population of PDAC patients at UICC TNM stage IIB is almost half of all patients, and PDAC patients at stage IIA and IIB were 79.5% of the total in this study. We have seen that for a small portion of patients at stage IIA and IIB, surgical removal of PDAC tumors leads to full recovery, while for most patients at stage IIA and IIB, disease recurrence and metastasis occur regardless of adjuvant therapy. In this study, there were no differences in postoperative survival times between PDAC patients at stage IIA and patients at stage IIB (Fig 5A), and the prognosis at stage IIA and IIB was relatively poor; the 3-year and 5-year survival rates at stage IIA and IIB were 29.3% and 21.0%, respectively (Table 7). The UICC TNM staging system determines the requirement for adjuvant therapy after surgical resection of PDAC tumors. Finding prognostic predictors that can discriminate PDAC patients with stage IIA and IIB into two prognosis groups (longer disease-free survival and/or better postoperative survival vs. shorter disease-free survival and/or poor postoperative survival) is necessary to consider suitable adjuvant treatment. As shown in Fig 5 and Table 8, the high immunohistochemical scores of the combination of PODXL with ITGB1 and the combination of BCL7B with ITGB1 effectively discriminated PDAC patients at stage IIA and IIB; postoperative median survival times of the high-expressing group of both PODXL and ITGB1 and the high-expressing group of both BCL7B and ITGB1 were relatively short compared to the corresponding low-expressing groups.

There was no significant correlation found between adjuvant therapy and PDAC patient prognosis in this study. It is notable that the combination of PODXL with ITGB1 and the combination of BCL7B with ITGB1 accurately predicted the postoperative outcomes of pancreatic cancer patients with or without adjuvant therapies. Several major adjuvant treatments including chemotherapy, chemoradiation, and chemotherapy plus chemoradiation have been used for more than thirty years. The JASPAC-01 trial performed in Japan showed that adjuvant oral fluoropyrimidine (S-1) chemotherapy improves overall survival compared to other chemotherapy regimens, including gemcitabine, and does not increase toxic side effects [21]. Adjuvant chemotherapy with S-1 is currently the standard care for resected PDAC in Japan. A clinical study (ESPAC-4) determined that adjuvant chemotherapy with gemcitabine plus capecitabine (an oral fluoropyrimidine) significantly increases overall survival compared with gemcitabine alone after resection of PDAC for Western patients [22]. A clinical study (CONKO-005) indicated that adjuvant gemcitabine plus erlotinib (an EGFR inhibitor) does not improve overall survival in patients with R0 PDAC resections [23]. A network meta-analysis reported that adding radiation to chemotherapy has no significant improvement on overall survival [24, 25]. On the other hand, a multicenter retrospective study reported that 5-year overall survival is 41.2% in postoperative PDAC patients (classified as T1-4, N0-1, or M0) treated with adjuvant chemoradiation compared with 25.7% in patients treated with adjuvant chemotherapy alone [26]. Since there is hope for adjuvant chemoradiation, further prospective studies should clarify if it is beneficial. Therefore, the development of adjuvant therapeutic approaches that are more beneficial than S-1 and gemcitabine plus capecitabine, which are currently the most effective adjuvant therapies for PDAC, is important to increase the survival rates of stage IIA-IIB PDAC patients. Since the present study indicated that postoperative prognosis of stage IIA-IIB PDAC patients with overexpression of both PODXL and ITGB1 and stage IIA-IIB PDAC patients with overexpression of both BCL7B and ITGB1 is extremely poor, immunohistochemical scores of PODXL and ITGB1 and those of BCL7B and ITGB1 could be used as reliable biomarkers of the response to adjuvant therapies prior to their initiation.

In conclusion, the combination of PODXL with ITGB1 and the combination of BCL7B with ITGB1 accurately predicted the postoperative prognosis of PDAC patients better than tumor size and the UICC TNM stage. The combination of PODXL with ITGB1 and the combination of BCL7B with ITGB1 can discriminate PDAC patients with worse prognosis at stage IIA-IIB. Patients with PDAC tumors that overexpress both PODXL and ITGB1 and/or both BCL7B and ITGB1 should be considered for suitable adjuvant treatment. When adjuvant therapeutic approaches that are more beneficial than S-1 and gemcitabine plus capecitabine are available, PDAC patients, especially those at stage IIA-IIB who are predicted to have worse prognosis, should be treated with more beneficial adjuvant therapies to increase the survival rate.

## Materials and methods

### Primary human PDAC samples

Resected PDAC tumor tissue was obtained from 102 patients during 1999–2014 at the Department of Surgery of Kochi Medical School Hospital (Nankoku, Japan) and Matsuyama Shimin Hospital (Matsuyama, Japan), as published previously [19]. No PDAC patients underwent neoadjuvant therapies. Of these patients, 86 had received adjuvant chemotherapy with gemcitabine or S-1, or radiation therapy or chemoradiation therapy after resection of PDAC. Postoperative follow-up consisted of physical examination, measurement of serum sialylated Lewis (a) blood group antigen (CA19-9), which is the clinical standard PDAC tumor biomarker, and computed tomography at 3- to 4-month intervals at Kochi Medical School Hospital and Matsuyama Shimin Hospital. Medical records of the 102 patients provided information regarding gender, age, tumor diameter, histology, UICC TNM stage, venous invasion, lymphatic invasion, and postoperative survival time. If PDAC patients died during follow-up, PDAC-related death was considered an outcome event. Observation was censored at PDAC-related death or end of observation. Follow-up to death or at least year 3 was 90% complete (92/102), and median follow-up in survivors was 64 months (interquartile range 32–91). Tumors were classified according to the Japanese Pancreas Society (JPS) classification [27] and the UICC TNM classification [28]. This study was approved by the ethical review boards of Kochi Medical School and Matsuyama Shimin Hospital prior to patient recruitment. Written informed consent was acquired from each patient prior to initiation.

### Immunohistochemical staining

Immunohistochemistry was performed using an anti-ITGB1 antibody (bs-0486R; Bioss, Woburn, MA) as published previously [10, 11]. The score of immunostaining was evaluated by two independent observers (SN and MF) who were blinded to the clinical and outcome data. The staining intensity was scored as: 1, weaker than the intensity of the surface staining of the islets of Langerhans; 2, equal to the intensity of the islets of Langerhans; 3, stronger than the intensity of the islets of Langerhans. The proportion of tumor cells was graded from 1 to 3: 1 (<50%), 2 (50–80%), and 3 (>80%). A total immunohistochemical score was calculated by summing the percentage score and the intensity score. The expression levels were classified into two groups based on the total score (low group, 2–3; high group, 4–6) with reference to previous reports [19, 29].

### Association analysis of ITGB1 between pancreatic tumor tissue and normal organ tissues

ITGB1 expressions between pancreatic tumor tissue and normal organ tissues including kidney, liver, lung, and pancreas were compared using MERAV [15].

### Statistical analysis

All statistical analyses were performed using R (version 3.3.3; The R Foundation, Wien, Austria) with the packages “KMsurv”, “rms”, and “survival” as published previously [19]. Fisher’s exact test and Chi-squared test were used to assess the correlation between ITGB1 expression levels and clinicopathological parameters. The analysis was timed to PDAC-related death. Factors as potential markers of prognostic significance included: age, gender, UICC TNM classification, degree of differentiation, lymphatic invasion, venous invasion, intrapancreatic nerve invasion, tumor size, and the immunohistochemical scores of PODXL, BCL7B, ARHGEF4, and ITGB1. We used arbitrary UICC stage categories (0, IA, IB vs. IIA and IIB vs. III and IV), clinical degree of differentiation categories (PanIN [30] and well-differentiated PDAC vs. moderately and poorly-differentiated PDAC), invasion strength categories (0 and 1 vs. 2 and 3), and tumor size (< 2 cm vs. 2.0 cm–3 cm vs. > 3.0 cm). Estimates of survival probabilities were performed by the Kaplan-Meier method. Univariate and multivariate analyses for the chosen explanatory variables were performed using the Cox proportional hazards (PH) regression model. Adjusted and unadjusted hazard ratios (aHR and HR) and 95% confidence intervals (95% CI) were given. The first prognostic model was chosen using a backwards and a forwards selection procedure for Akaike’s Information Criterion (AIC) with the factors as potential markers. Martingale residuals were used to evaluate nonlinearity for age. Tests based on Schoenfeld residuals were used to evaluate violations of the assumption of PH in the multivariate models [31]. By testing of the assumption PH, we used a second model with factors of the first model and interactions for intrapancreatic nerve invasion and the immunohistochemical scores of PODXL, BCL7B, ARHGEF4, and ITGB1. *P* values < 0.05 were considered significant and are indicated by asterisks in the figures.

## Supporting information

S1 Table Multivariate analysis using the Cox proportional hazards regression model. (DOCX)

S2 Table Data of adjuvant therapy, prognosis, TNM stage, and immunostaining score of ITGB1 in 102 patients with pancreatic cancer. (DOCX)

S1 Fig Correlation between adjuvant therapy and prognosis in PDAC patients. Kaplan-Meier analysis of postoperative survival according to adjuvant chemotherapy is shown.
(TIF)
