# Supplementary material for: Overexpression of PODXL/ITGB1 and BCL7B/ITGB1 accurately predicts unfavorable prognosis compared to the TNM staging system in postoperative pancreatic cancer patients
Source: PLoS One. 2019 Jun 5;14(6):e0217920. doi: 10.1371/journal.pone.0217920 (PMC6550449; doi:10.1371/journal.pone.0217920)
Supplement: S1 Table — (DOCX) [file pone.0217920.s001.docx]

**S1 Table.** Multivariate analysis using the Cox proportional hazards regression model

|  | HR (95% CI) | P |
| --- | --- | --- |
| **Stage*** |  |  |
| 0, IA, IB | 0.25 (0.09-0.70) | 0.009 |
| IIA, IIB | Reference |  |
| III, IV | 3.05 (1.25-7.42) | 0.014 |
| **Adjuvant therapy**  Surgery |  |  |
| Radiation | 0.59(0.14-2.39) | 0.457 |
| Chemotherapy | 0.85(0.49-1.46) | 0.545 |
| Chemoradiation | Reference |  |
| None | 1.19(0.57-2.47) | 0.649 |
| **ARHGEF4 expression** | 2.52 (1.28-5.00) | 0.007 |
| **ARHGEF4 expression and intrapancreatic nerve invasion** | 2.97 (1.36-6.49) | 0.006 |
| **ARHGEF4 and ITGB1 expression** | 0.22 (0.08-0.59) | 0.003 |
| **PODXL and ITGB1 expression** | 6.27 (2.58-15.2) | < 0.001 |
| **BCL7B and ITGB1 expression** | 3.93 (1.74-8.91) | 0.001 |

*, Classified according to the classification of International Union against Cancer.
