# Supplementary material for: Overexpression of PODXL/ITGB1 and BCL7B/ITGB1 accurately predicts unfavorable prognosis compared to the TNM staging system in postoperative pancreatic cancer patients
Source: PLoS One. 2019 Jun 5;14(6):e0217920. doi: 10.1371/journal.pone.0217920 (PMC6550449; doi:10.1371/journal.pone.0217920)
Supplement: S2 Table — (DOCX) [file pone.0217920.s002.docx]

**S2 Table.** Data of adjuvant therapy, prognosis, TNM stage, and immunostaining score of ITGB1 in 102 patients with pancreatic cancer

| **No.** | **Adjuvant therapy** | **Status** | **Survival time (M)** | **UICC Stage** | **ITGB1 score** |
| --- | --- | --- | --- | --- | --- |
| 1 | Chemoradiation | Dead | 24 | IIB | 3 |
| 2 | Chemoradiation | Dead | 84 | IIA | 3 |
| 3 | Chemoradiation | Dead | 125 | IIB | 3 |
| 4 | Chemoradiation | Dead | 59 | IB | 3 |
| 5 | Chemoradiation | Dead | 29 | IIA | 3 |
| 6 | Chemoradiation | Dead | 41 | IIB | 3 |
| 7 | Chemoradiation | Dead | 16 | IIB | 4 |
| 8 | Chemoradiation | Dead | 15 | IIB | 3 |
| 9 | Chemoradiation | Dead | 12 | IIA | 4 |
| 10 | Chemoradiation | Dead | 25 | IIA | 2 |
| 11 | Chemoradiation | Alive | 100 | IIB | 3 |
| 12 | Chemoradiation | Dead | 23 | IIB | 3 |
| 13 | Chemoradiation | Dead | 10 | IIB | 3 |
| 14 | Chemoradiation | Dead | 27 | IIA | 4 |
| 15 | Chemotherapy | Alive | 89 | IIA | 3 |
| 16 | Chemotherapy | Dead | 13 | IIA | 4 |
| 17 | Chemotherapy | Dead | 14 | IIB | 4 |
| 18 | None | Alive | 83 | IA | 3 |
| 19 | Chemotherapy | Dead | 21 | IIB | 4 |
| 20 | Chemotherapy | Dead | 6 | IIA | 4 |
| 21 | Chemotherapy | Dead | 35 | IIB | 3 |
| 22 | Chemotherapy | Dead | 60 | IIB | 3 |
| 23 | None | Dead | 14 | IA | 4 |
| 24 | Chemoradiation | Dead | 20 | IIB | 3 |
| 25 | Chemoradiation | Alive | 74 | IIB | 3 |
| 26 | Chemoradiation | Dead | 13 | IIB | 2 |
| 27 | Chemoradiation | Dead | 13 | IIA | 4 |
| 28 | Chemotherapy | Dead | 25 | IIA | 3 |
| 29 | Chemoradiation | Dead | 7 | IIB | 2 |
| 30 | Chemoradiation | Dead | 35 | IIB | 3 |
| 31 | Chemoradiation | Dead | 12 | IIB | 4 |
| 32 | Chemoradiation | Dead | 10 | IIA | 4 |
| 33 | Chemoradiation | Dead | 23 | IA | 3 |
| 34 | Chemotherapy | Dead | 33 | IIA | 4 |
| 35 | Chemoradiation | Dead | 25 | IIB | 3 |
| 36 | Chemoradiation | Alive | 45 | IIA | 3 |
| 37 | Chemoradiation | Dead | 26 | IIB | 3 |
| 38 | Chemoradiation | Dead | 20 | IIA | 3 |
| 39 | Chemotherapy | Dead | 33 | IIA | 3 |
| 40 | Chemotherapy | Dead | 9 | IIB | 3 |
| 41 | Chemoradiation | Alive | 32 | IA | 4 |
| 42 | Chemotherapy | Dead | 11 | IIA | 4 |
| 43 | Chemoradiation | Dead | 24 | IIB | 3 |
| 44 | Chemotherapy | Dead | 13 | IIB | 4 |
| 45 | Chemotherapy | Dead | 8 | IIB | 3 |
| 46 | Chemoradiation | Alive | 34 | IIB | 3 |
| 47 | Chemoradiation | Dead | 29 | IIB | 3 |
| 48 | Chemotherapy | Alive | 35 | IIB | 4 |
| 49 | Chemoradiation | Dead | 39 | IIB | 3 |
| 50 | Chemotherapy | Dead | 13 | IIA | 4 |
| 51 | Chemotherapy | Alive | 89 | IB | 3 |
| 52 | Chemoradiation | Alive | 84 | IIB | 3 |
| 53 | Chemotherapy | Dead | 12 | III | 3 |
| 54 | None | Dead | 22 | IIB | 4 |
| 55 | Chemoradiation | Dead | 15 | IIB | 4 |
| 56 | Chemoradiation | Alive | 41 | IIB | 3 |
| 57 | None | Dead | 7 | IIB | 4 |
| 58 | Chemotherapy | Alive | 18 | IIB | 4 |
| 59 | Chemotherapy | Alive | 19 | IIA | 3 |
| 60 | Chemotherapy | Alive | 20 | IB | 4 |
| 61 | Chemotherapy | Alive | 22 | IIB | 3 |
| 62 | Radiation | Alive | 24 | IIB | 4 |
| 63 | Chemotherapy | Alive | 26 | IIA | 3 |
| 64 | Chemotherapy | Dead | 15 | IIB | 4 |
| 65 | Chemotherapy | Alive | 27 | IIB | 3 |
| 66 | Chemoradiation | Dead | 20 | IIA | 3 |
| 67 | Chemotherapy | Dead | 21 | IIB | 4 |
| 68 | Chemotherapy | Alive | 41 | IB | 3 |
| 69 | Chemotherapy | Alive | 44 | IB | 3 |
| 70 | Chemotherapy | Dead | 19 | IIA | 3 |
| 71 | Chemotherapy | Dead | 10 | IIA | 4 |
| 72 | Chemotherapy | Alive | 64 | IIA | 3 |
| 73 | Chemotherapy | Dead | 18 | IV | 3 |
| 74 | Chemotherapy | Dead | 9 | IIB | 3 |
| 75 | Chemotherapy | Dead | 29 | IIA | 3 |
| 76 | None | Alive | 78 | 0 | 2 |
| 77 | Chemotherapy | Dead | 40 | IIB | 3 |
| 78 | Chemotherapy | Alive | 91 | IIB | 4 |
| 79 | Chemotherapy | Dead | 17 | IIA | 3 |
| 80 | Chemotherapy | Alive | 96 | IIB | 4 |
| 81 | Chemoradiation | Dead | 13 | IV | 4 |
| 82 | None | Alive | 101 | IIA | 3 |
| 83 | Radiation | Dead | 39 | III | 2 |
| 84 | Chemotherapy | Alive | 107 | 0 | 3 |
| 85 | Chemotherapy | Dead | 18 | IIB | 3 |
| 86 | Chemotherapy | Dead | 26 | IIB | 3 |
| 87 | Chemotherapy | Dead | 36 | IIB | 3 |
| 88 | None | Alive | 136 | IIA | 3 |
| 89 | None | Dead | 50 | IIA | 3 |
| 90 | Chemotherapy | Alive | 138 | IB | 2 |
| 91 | None | Dead | 5 | IV | 4 |
| 92 | Radiation | Dead | 67 | IIA | 2 |
| 93 | Radiation | Dead | 23 | IIB | 2 |
| 94 | None | Dead | 35 | IB | 2 |
| 95 | Chemotherapy | Dead | 27 | IIB | 3 |
| 96 | None | Dead | 7 | IIB | 4 |
| 97 | None | Dead | 10 | IIB | 3 |
| 98 | None | Dead | 33 | IIA | 5 |
| 99 | Chemotherapy | Dead | 13 | IV | 4 |
| 100 | None | Dead | 23 | IIA | 3 |
| 101 | None | Dead | 24 | IIA | 3 |
| 102 | None | Alive | 192 | IB | 3 |
