# Supplementary figures and images for: Overexpression of PODXL/ITGB1 and BCL7B/ITGB1 accurately predicts unfavorable prognosis compared to the TNM staging system in postoperative pancreatic cancer patients
Source: PLoS One. 2019 Jun 5;14(6):e0217920. doi: 10.1371/journal.pone.0217920 (PMC6550449; doi:10.1371/journal.pone.0217920)

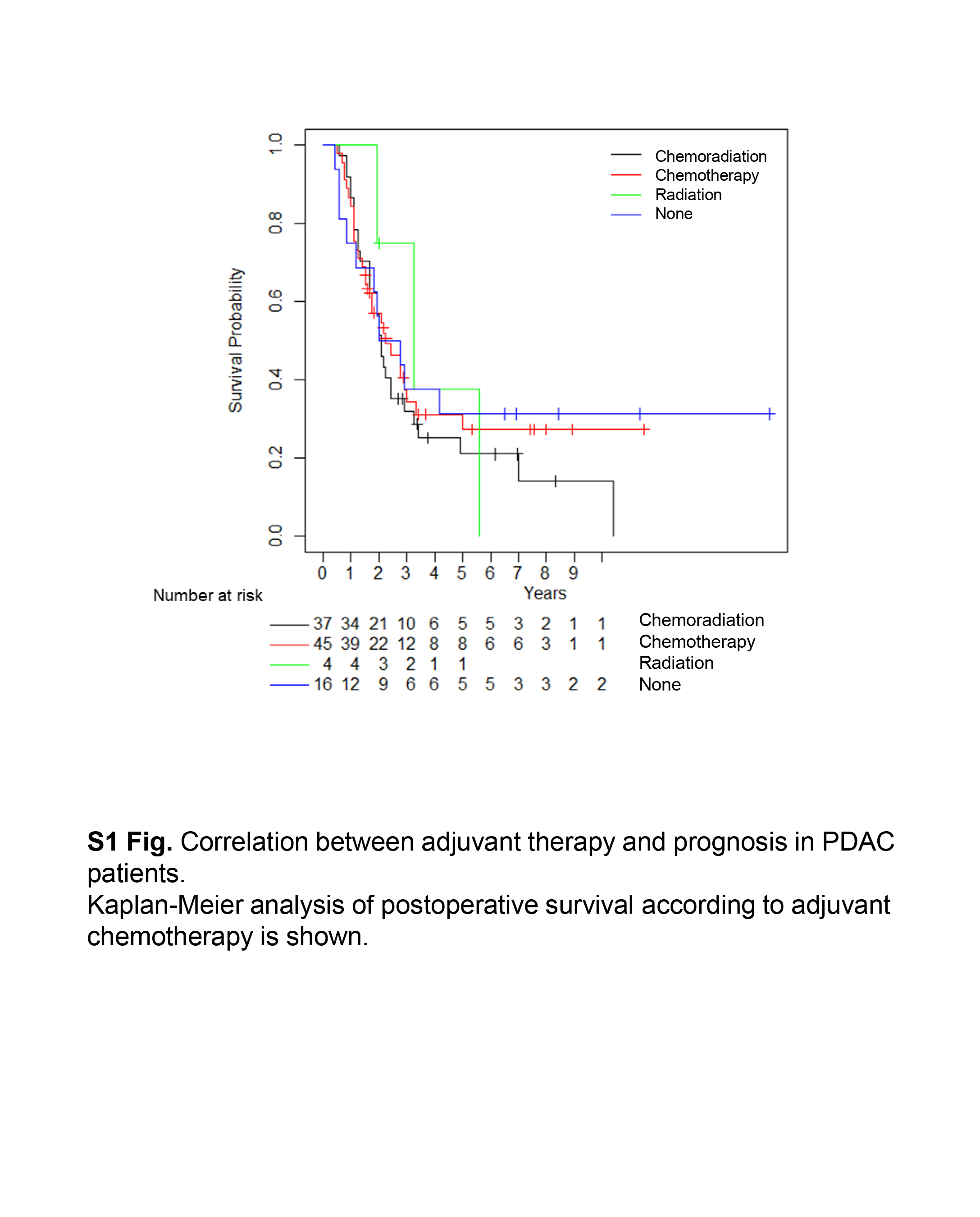

Supplement: S1 Fig — Kaplan-Meier analysis of postoperative survival according to adjuvant chemotherapy is shown. (TIF) [file pone.0217920.s003.tif]
